# Displacement Sensing of an Active String Actuator Using a Step-Index Multimode Optical Fiber Sensor †

**DOI:** 10.3390/s22093232

**Published:** 2022-04-22

**Authors:** Weihang Tian, Shuichi Wakimoto, Takefumi Kanda, Daisuke Yamaguchi

**Affiliations:** Graduate School of Natural Science and Technology, Okayama University, Okayama 700-8530, Japan; pd339tfj@s.okayama-u.ac.jp (W.T.); kanda-t@okayama-u.ac.jp (T.K.); yamaguchi20@okayama-u.ac.jp (D.Y.)

**Keywords:** thin artificial muscle, active string actuator, step-index multimode optical fiber, displacement sensing

## Abstract

A thin McKibben artificial muscle is a pneumatic actuator with an outer diameter of only 1.8 mm. We fabricated a string-shaped actuator called an “active string actuator,” which achieves a high contractile displacement by accumulating thin McKibben artificial muscles. To control the displacement, the length of the active string actuator should be estimated. However, this is difficult because bulky and rigid sensors are unsuitable for the sensor element of the active string actuator. Therefore, in this study, we propose a new sensing method for estimating the length of an active string actuator. The proposed sensing system is simple and comprises only three components: a step-index multimode optical fiber, a light emitter, and a light receiver. A step-index multimode optical fiber was combined with the active string actuator, and the length was estimated from the change in the amount of light propagating in the optical fiber when the active string actuator was driven. Fundamental experiments were conducted in this study, and the results demonstrated that the optical fiber sensor value changed with the actuator length. This suggests that it is possible to estimate the displacement of an active string actuator using an optical fiber sensor.

## 1. Introduction

The McKibben artificial muscle is a pneumatic actuator developed in the 1950s [1]. The artificial muscle contracts axially and expands radially. Contraction displacement is generally utilized as the mechanical output. It has a simple structure consisting of a rubber tube and sleeve fiber and is characterized by good human compatibility and a high power-to-weight ratio. In recent years, general-purpose power-assist and rehabilitation devices that incorporate McKibben artificial muscles have become commercially available [2,3].

One of the authors of this paper developed a thin McKibben artificial muscle (hereinafter referred to as a thin artificial muscle) with an outer diameter of only 1.8 mm. By contrast, the outer diameter of a commercially available McKibben artificial muscle is between 10 mm and several tens of millimeters. The outer diameter of the developed thin artificial muscle is only 1.8 mm, making it more compliant than conventional McKibben artificial muscles. Therefore, it is possible to consider it to be composed of fibers and accumulate them in the form of strings and cloths to realize accumulated structures with softness as well as high contractility and force [4,5,6,7,8]. They are not only applied to power-assist and rehabilitation devices, but also to musculoskeletal mechanisms that mimic the redundant drive mechanism of the human body and robot arms with multiple degrees of freedom for contraction, bending, and twisting movements [9,10,11,12,13].

In a previous study, we fabricated a string-shaped actuator called an “active string actuator” by accumulating thin artificial muscles using a string production process, and it was confirmed that the generated force and contraction ratio were improved [14]. To control the displacement, it is necessary to sense the length of the actuator. However, conventional sensors such as potentiometers or encoders are not suitable for the sensor elements of the active string actuator because they are bulky and rigid. For instance, when the active string actuator is applied to a power-assist device, conventional sensors can impede user movements owing to their weight and rigidity. Therefore, an appropriate displacement sensing method that agrees with the future of active string actuators is required so that the advantages of the actuator are retained.

Several studies have been conducted to sense the displacement of “soft” actuators such as the active string actuator. The sensors of electrical resistance types, conductive rubber sensors [15], and the liquid conductor sensors (eutectic gallium indium, eGaIn) [16] have been applied to soft actuators. Functional fibers such as sleeve fibers, which are part of the McKibben artificial muscle, can be used to estimate the displacement of the artificial muscle based on the change in the inductance [17] or capacitance [18]. In addition, Hall effect sensors [19], optical sensors [20,21], and ultrasonic sensors [22] have been used to measure the displacement of artificial muscles. In many sensing methods, the sensor itself has a complex structure that is difficult to fabricate and its integration in the actuator requires a complex procedure. Moreover, using a rigid sensor in the drive unit of the actuator may compromise the flexibility of the soft actuator [19,20,21,22].

In this study, we propose a new sensing application for estimating the displacement of an active string actuator while avoiding the aforementioned problems. A step-index multimode optical fiber was integrated in the active string actuator, and the length was estimated based on the change in the amount of light propagating in the optical fiber when the active string actuator was driven. The advantages of this sensing application are as follows:

1. Simple structure: The sensing system comprises only three components, namely, an optical fiber, a light emitter, and a light receiver. Almost all of the components are commercially available, making them inexpensive and easy to manufacture.
2. Flexible: The light emitter and receiver are located outside the drive unit, and the optical fiber located in the drive unit is flexible and thin.

We believe that the active string actuator with the optical fiber sensor that we have developed will facilitate the development of rehabilitation equipment and lead to the development of safer and more effective equipment due to its flexibility and displacement estimation capabilities. Furthermore, it will be useful in a wide variety of applications where soft robotics abilities are required. 

In this paper, the structure and driving principle of an active string actuator are first introduced. Then, the sensing principle of the proposed optical fiber sensor and its integration in the active string actuator are explained, and the experimental system developed to evaluate the constructed sensor is presented in Section 2. The actuation and sensing characteristics of the active string actuator with the optical fiber sensor are described in Section 3, and the conclusions and future work are presented in Section 4.

## 2. Materials and Methods

### 2.1. Thin Artificial Muscles and Active String Actuator

**Figure 1:**
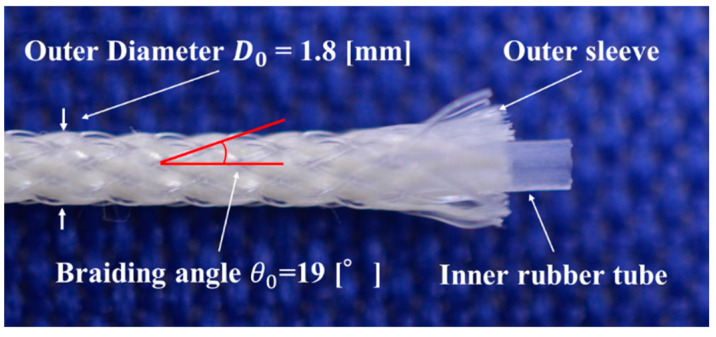
Appearance and structure of the thin artificial muscle.

An active string actuator was realized by accumulating thin artificial muscles in a round string structure. Figure 1 shows the appearance and structure of the thin artificial muscle. It has an outer diameter of 1.8 mm and consists of an inner silicone rubber tube and outer sleeve braiding of 24 fibers. 

The theoretical construction force *F* of the McKibben artificial muscle is calculated using the equation proposed by Schulte [23,24] as follows:

(1) $$F=\frac{\pi }{4}D_{0}{}^{2}P{\left(\frac{1}{\sin\theta_{0}}\right)}^{2}\left\{3{\left(1-\varepsilon \right)}^{2}\cos^{2}\theta_{0}-1\right\},$$

where $D_{0}$ is the initial diameter of the rubber tube, P is the applied pressure, $\theta_{0}$ is the initial braiding angle of the sleeve, and *ε* is the contraction ratio. Equation (1) indicates that the construction force *F* depends on the initial braiding angle $\theta_{0}$. Additionally, it is defined as half of the angle between the fibers of the sleeve, as shown in Figure 1. The braiding angle of the thin artificial muscles used in this study is 19°.

**Figure 2:**
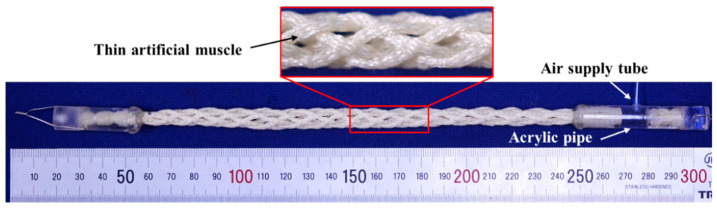
Appearance and structure of the active string actuator.

**Figure 3:**
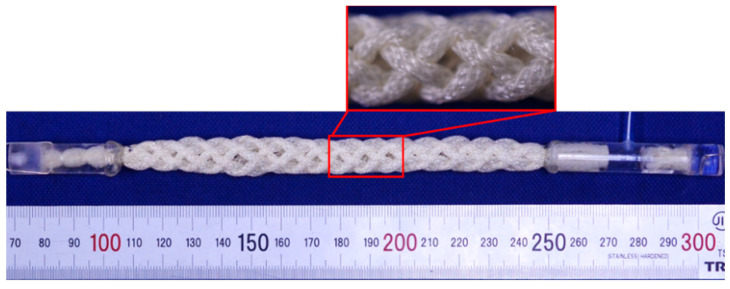
Active string actuator with air pressure of 0.3 MPa.

Eight thin artificial muscles were assembled into a string shape using a string production process. One side was sealed and the other side was connected to an acrylic pipe resembling an air supply tube, as shown in Figure 2. The eight artificial muscles were pressurized together by applying pneumatic pressure to the air supply tube. In this study, the actuator made using this approach is called an “active string actuator.” Figure 3 the condition shows the structure of the active string actuator under the application of a pneumatic pressure of 0.3 MPa. Compared with the initial condition shown in Figure 2, the actuator contracts axially.

**Figure 4:**
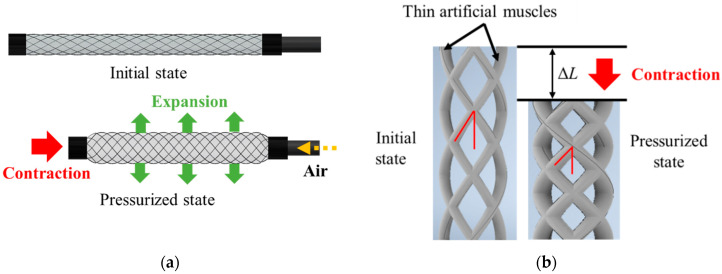
Contraction of the active string actuator: (**a**) contraction of the thin artificial muscle; (**b**) contraction using the pantograph mechanism.

When pneumatic pressure is applied to the thin artificial muscle, it expands in the radial direction and contracts in the axial direction, as shown in Figure 4a, and the total length of the active string actuator decreases. When the artificial muscles were assembled in the form of a braided string, the crossing angle of the thin artificial muscles increased with the application of air pressure, as shown in Figure 4b, which caused the active string actuator to contract, as it functions similar to the pantograph mechanism. The contraction of the active string actuator is achieved by combining the contraction of the thin artificial muscle and the pantograph mechanism, such that the entire active string actuator significantly contracts in the axial direction.

### 2.2. Step-Index Multimode Optical Fiber Sensor

A step-index multimode optical fiber (GCK–20E 0.5, MITUBISHI CHEMICAL) was used in this study. The optical fiber has the advantages of low cost, flexibility, bending resistance, and small diameter, and it can be easily integrated in active string actuators. The method for integrating optical fibers in the active string actuator is described in Section 2.3. 

**Figure 5:**
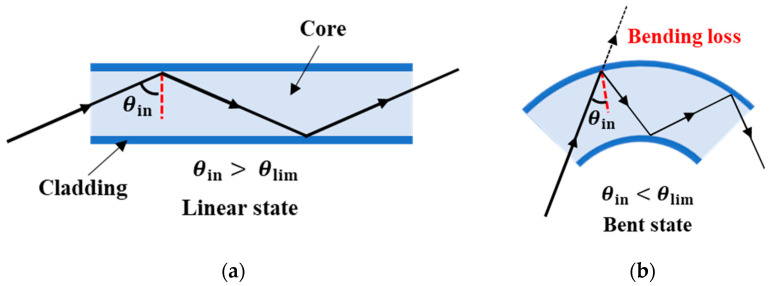
Bending loss of the optical fiber: (**a**) linear state; (**b**) bent state.

The outer diameter of the step-index multimode optical fiber used in this study is 0.5 mm, and the fiber comprises two layers: a core layer and cladding layer. The refractive index of the cladding layer is lower than that of the core layer. When the light incident angle $\theta_{\mathrm{in}}$ is greater than the critical angle $\theta_{\lim}$, the incident light in the optical fiber propagates through the core layer with repeated total reflection, as depicted in Figure 5a. However, when the optical fiber is bent, the incident angle $\theta_{\mathrm{in}}$ becomes smaller than the critical angle $\theta_{\lim},$ and the light begins to leak out as shown in Figure 5b. This phenomenon is known as the bending loss of light in the optical fiber. Consequently, as the radius of curvature of the optical fiber decreases, the amount of light propagating in the optical fiber decreases accordingly. 

**Figure 6:**
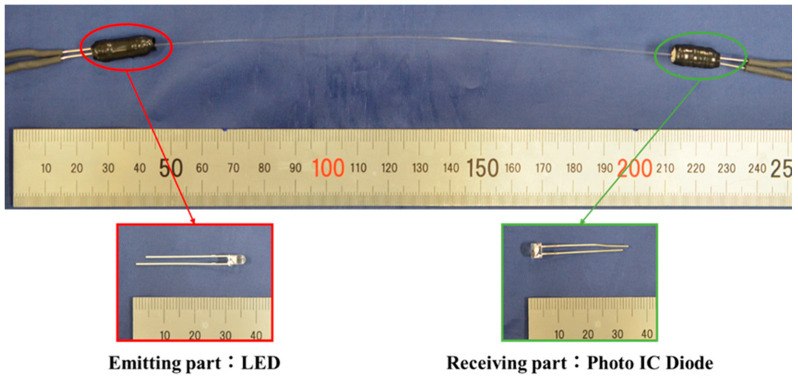
Appearance and structure of the step-index multimode optical fiber sensor.

To use a step-index multimode optical fiber as a sensor, a light emitter is required at one end and a light receiver at the other end to measure the light intensity. As shown in Figure 6, we used a white LED (C503D-WAN-CCbEb151, Cree LED) as the light emitter and a photo IC diode (S13948-01SB, Hamamatsu Photonics) as the light receiver. A photo IC diode is an electronic component that generates an electrical current output in proportion to the amount of light incident on it. In this study, the photo IC diode was connected in series to a resistor, and the voltage output of the resistor was used as the sensor voltage.

**Figure 7:**
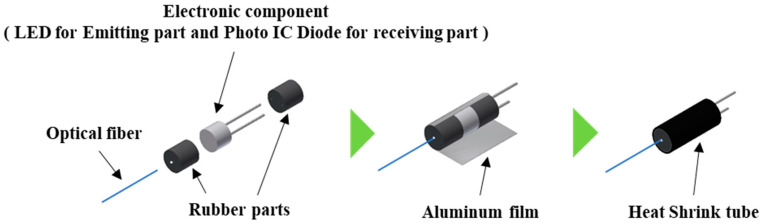
Structure of the emitting and receiving parts.

As shown in Figure 7, rubber parts with small openings of 0.5 mm in diameter were fabricated, and electronic components (LED for the light-emitting part and photo IC diode for the light-receiving part) are sand by them. One of the rubber parts had an opening for inserting the optical fiber, and the other had two small openings for electrical wiring. They were covered with a thin aluminum film and heat-shrinkable tubes to prevent penetration of external light and light leakage from the components.

**Figure 8:**
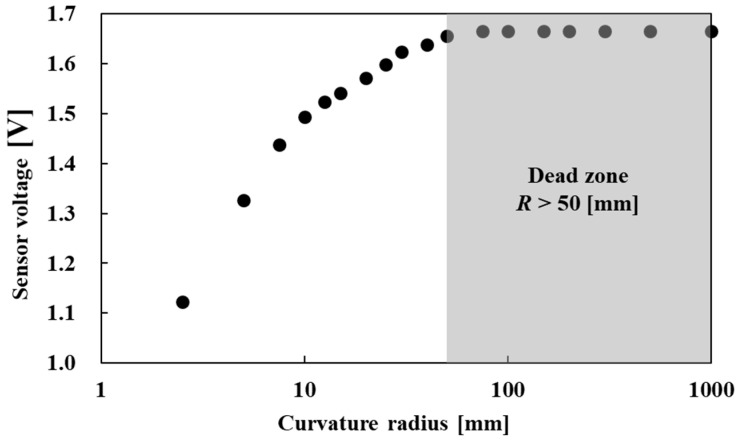
Relationship between the curvature radius and sensor voltage.

**Figure 9:**
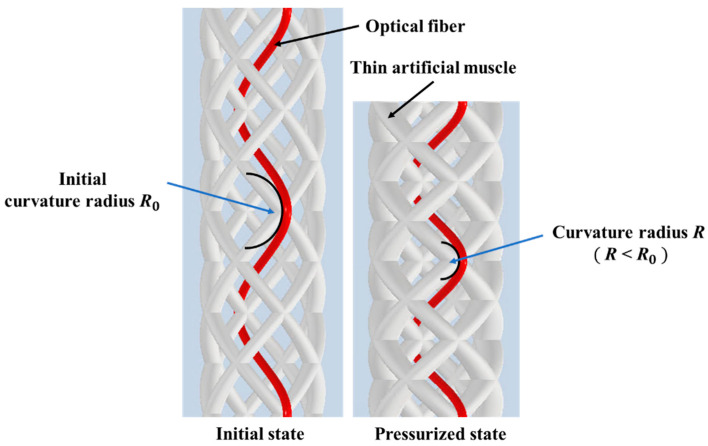
Integrating the optical fiber sensor in the active string actuator.

We measured the change in the sensor output corresponding to the curvature of a single optical fiber. Figure 8 illustrates the experimental setup. We connected the light emitter to one end of the optical fiber and the light receiver to the other end. With the optical fiber curving, the radius of curvature of the optical fiber and the sensor output, which is the voltage of the receiving part, were recorded. Figure 9 shows the relationship between the radius of curvature of the optical fiber and the sensor output. Although the sensor output changed corresponding to the radius of curvature with a range lower than 50 mm, no change was observed in the sensor output for a radius of curvature greater than 50 mm. The reason for this sensor dead zone is that the light incident angle $\theta_{\mathrm{in}}$ is smaller than $\theta_{\lim}$ when the bending extent is low.

### 2.3. Active String Actuator with Optical Fiber Sensor

The optical fiber sensor itself uses an already known phenomenon, the bending loss, but as a means of estimating the displacement of an active string actuator has not been available before, it is new as an actuator-sensor system.

The optical fiber was integrated in the active string actuator in a wave-like form by sewing between braided thin artificial muscles, as depicted in Figure 9. When the active string actuator contracted owing to air pressure, the waveform of the optical fiber changed with a change in the radius of curvature. Consequently, the amount of light propagating in the optical fiber decreased. The length of the active string actuator can be estimated by measuring the amount of light using a photo IC diode.

**Figure 10:**
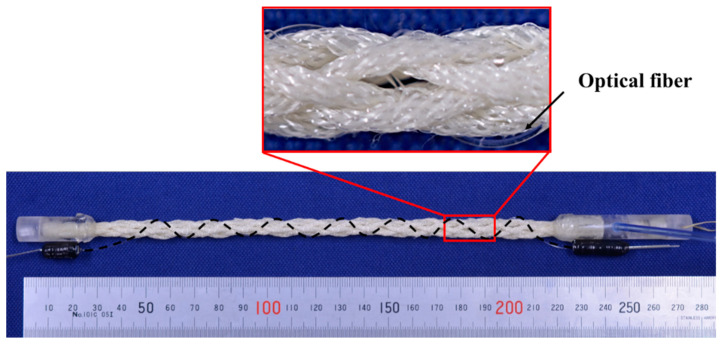
Appearance of the active string actuator that integrates the optical fiber sensor in a wave-like pattern.

Figure 10 shows the appearance of the active string actuator with the optical fiber sensor. The black dashed line represents the optical fiber embedded in braided thin artificial muscles. 

**Figure 11:**
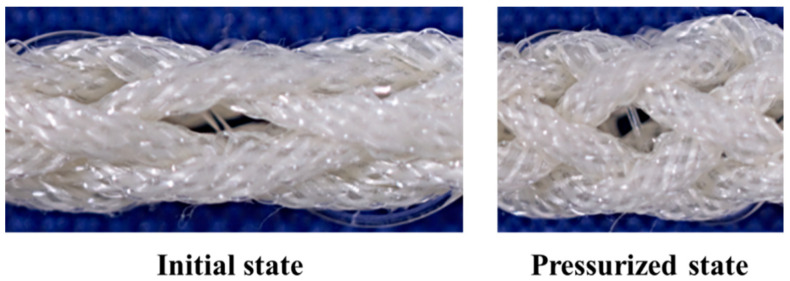
Enlarged view of the active string actuator.

Figure 11 shows the optical fiber in the initial state and contraction state, wherein air pressure was applied. The radius of curvature of the optical fiber decreased with the contraction of the active string actuator.

### 2.4. Experimental System

**Figure 12:**
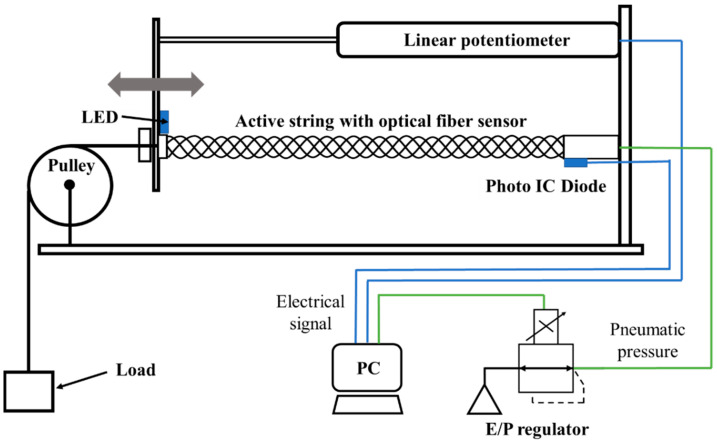
Schematic of the measurement system.

Figure 12 illustrates the experimental setup for measuring the basic characteristics of the active string actuator with the optical fiber. To evaluate the optical fiber sensor, we used a linear potentiometer (LP-100FJ-5K, MIDORI PRECISIONS) to measure the actual length of the active string actuator. The signal from the linear potentiometer and photo IC diode was read using a PC. Additionally, an electropneumatic regulator (EVT500-0-E2-3, CKD) was used to apply air pressure proportional to the output signal from the PC to the active string actuator.

## 3. Results and Discussion

### 3.1. Contraction Characteristics of the Active String Actuator

The contraction capabilities of the active string actuator with and without the optical fiber were measured to examine the effect of the optical fiber sensor on the actuation characteristics of the active string actuator. In the measurement experiments, the load was set to 1.0 N to ensure that the active string actuator was taut and driven stably. Air pressure was continuously applied to the active string actuator from 0.0 MPa to 0.3 MPa (with and without the optical fiber) and then reduced to 0.0 MPa. 

**Figure 13:**
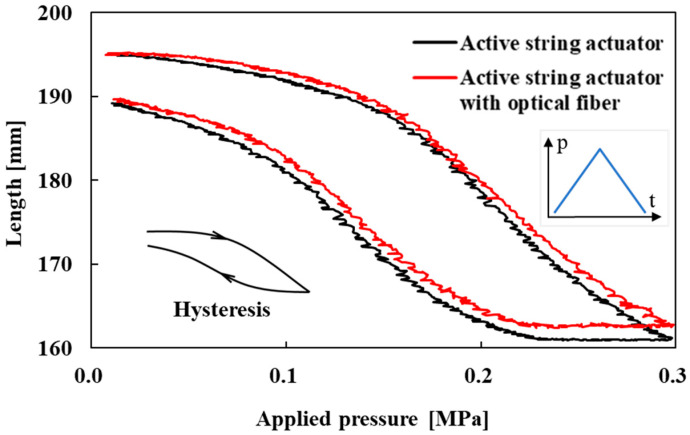
Contraction characteristics of the active string actuator with and without the optical fiber sensor.

Figure 13 shows the contraction characteristics of the active string actuator with and without the optical fiber. The red and black lines in the figure indicate these characteristics. This can be observed from the figure in which hysteresis occurs. The generation of hysteresis in the driving characteristics of the accumulated structure of thin artificial muscles is a general phenomenon and has been reported [6,25]. There are two main reasons for this: the effect of the original hysteresis of the thin artificial muscles and the generation of friction force between the thin artificial muscles.

At an applied pressure of 0.3 MPa, the contraction of the active string actuator was 34.1 mm, whereas that of the active string actuator with the optical fiber was 32.6 mm. By embedding the optical fiber, the contraction was reduced by 1.5 mm of the absolute value (this represents 4.5% of the maximum contraction of the active string actuator without the optical fiber). The stiffness of the optical fiber (although it is not a rigid material) and contact friction between the optical fiber and thin artificial muscles inhibit the contraction of the active string actuator. The rate of contraction can be reduced by increasing the composite pitch and using low-stiffness optical fibers (thin optical fibers).

The reduction rate can be decreased using an optical fiber that is thinner than the one used in this study and by optimizing the pitch of the wave pattern when it is combined.

### 3.2. Characteristics of the Active String Actuator with the Optical Fiber Sensor

**Figure 14:**
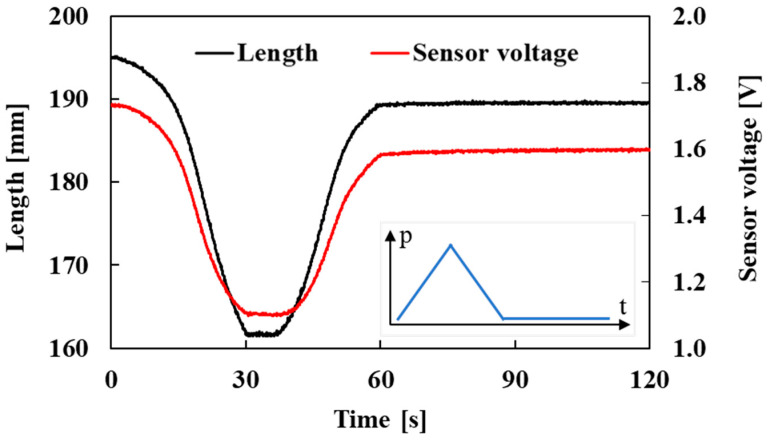
Length and sensor voltage variation with respect to time (pneumatic pressure: 0.0 MPa → 0.3 MPa → 0.0 MPa, then held until 120 s).

The correlation between the sensor voltage and the length of the active string actuator with the optical fiber sensor was investigated. In the experiment, the load was set to 1.0 N as in the experiment in Section 3.1. Pneumatic pressure was repeatedly applied to the active string actuator with the optical fiber sensor from 0.0 MPa to 0.3 MPa for 30 s and then reduced to 0.0 MPa for 30 s. To test the robustness of the sensor, the pressure was reduced to 0.0 MPa and held for 60 s. The changes in the length of the active string actuator and the voltage output of the optical fiber sensor over time are shown in Figure 14. 

Figure 14 shows the change in the sensor voltage and length; the red and black lines indicate these characteristics. When the air pressure was held at 0.0 MPa (60 s to 120 s), the sensor output was stable.

**Figure 15:**
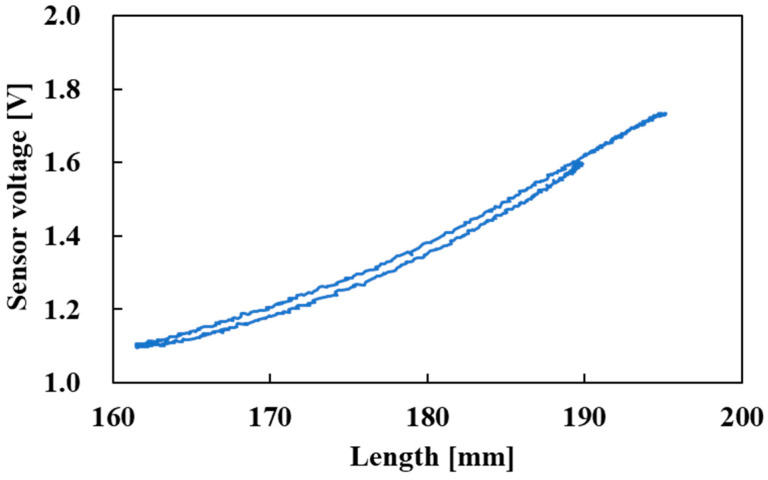
Relationship between length and sensor voltage (pneumatic pressure: 0.0 MPa → 0.3 MPa → 0.0 MPa, then held until 120 s).

The relationship between the length of the active string actuator and the voltage output of the optical fiber sensor is shown in Figure 15. The hysteresis was slight, and the sensor voltage correlated with the length of the active string actuator over the entire driving range. Since the optical fiber was not fixed to thin artificial muscles, this was the cause of the hysteresis.

**Figure 16:**
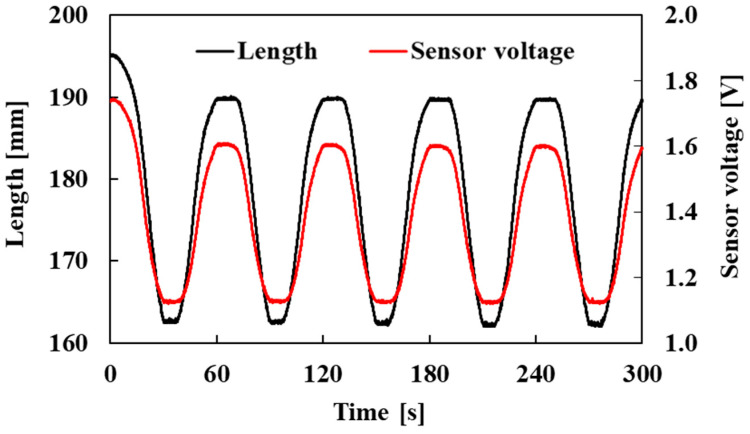
Length and sensor voltage variation with respect to time (pneumatic pressure: 0.0 MPa → 0.3 MPa → 0.0 MPa, five times).

In addition, to check the reproducibility of the sensor output, pneumatic pressure was repeatedly applied to the active string actuator with the optical fiber sensor five times, rising from 0.0 MPa to 0.3 MPa and falling back to 0.0 MPa. The changes in the length of the active string actuator and the voltage output of the optical fiber sensor over time are shown in Figure 16. Notably, the electrical signal from the PC in Figure 12 is a triangular waveform; however, the actuator length changed sinusoidally, corresponding to the pneumatic pressure because of its nonlinearity.

Figure 16 shows the change in the sensor voltage and length; the red and black lines indicate these characteristics. As is evident, the sensor voltage followed the length without a phase shift.

**Figure 17:**
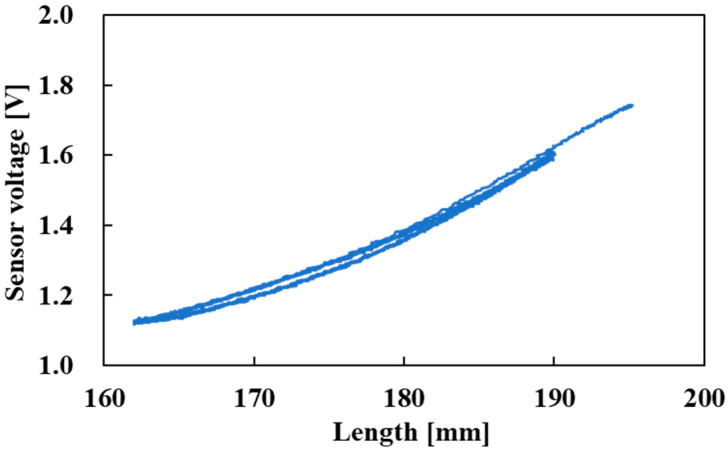
Relationship between length and sensor voltage (pneumatic pressure: 0.0 MPa → 0.3 MPa → 0.0 MPa, five times).

The relationship between the length of the active string actuator and voltage output of the optical fiber sensor is shown in Figure 17. The relationship between the active string length and the sensor voltage followed the same route, so we consider the sensor output to be highly reproducible.

**Figure 18:**
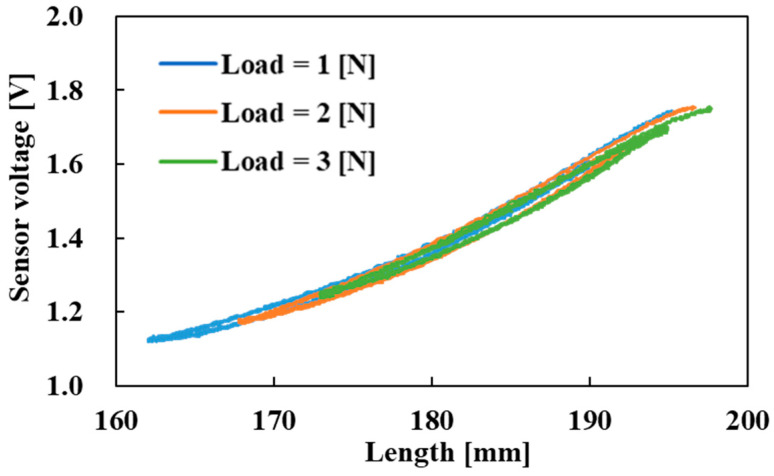
Change in sensor voltage due to load.

Furthermore, experiments were conducted in which loads of 2.0 N and 3.0 N were added to the actuator to examine the effect of load on the sensor characteristics. The condition for pneumatic pressure was the same as that in the experiment in Figure 16. Pneumatic pressure was repeatedly applied to the active string actuator with the optical fiber sensor five times, rising from 0.0 MPa to 0.3 MPa and falling back to 0.0 MPa. The measurement results are shown in Figure 18. The figure shows a comparison of three conditions, namely, loads of 1.0 N, 2.0 N, and 3.0 N.

The driving range of the actuator (i.e., length range) depends on the load; therefore, the length range differs according to the load. However, it was found that the sensor voltage of each condition overlapped. This implies that the sensor output corresponds to the length of the active string actuator and does not depend on the load. The sensor voltage correlated with the length of the active string actuator in the entire driving range, and the relationship between the length L and the sensor voltage $V_{S}$ derived from Figure 18 is approximated as given below. Here, R² is the coefficient of determination of the approximate equation; a value of 0.993 demonstrates the high agreement of the equation.

(2) $$L=-41.89V_{S}{}^{2}+170.7V_{S}+24.95$$

R² = 0.993.

The maximum error in the length between the measurement results and the approximate formula is 2.5 mm, which represents 7.6% of the driving range of the actuator. The optical fiber was not fixed to thin artificial muscles, so hysteresis occurred. Although currently the error may still be large, through this study, the voltage output of the optical fiber sensor was correlated with the length of the active string actuator, indicating the possibility of estimating the length of the active string actuator by using the optical fiber sensor.

## 4. Conclusions

In this study, we propose a new method for sensing the length of an active string actuator. We integrated a step-index multimode optical fiber in an active string actuator and used a photo IC diode to measure changes in the amount of light propagating in the optical fiber to estimate the length of the active string actuator. The step-index multimode optical fiber can be easily integrated in active string actuators in a wave-like pattern. Fundamental experiments were conducted, and the results are summarized below.

1. The integration of the optical fiber sensor reduced the contraction of the active string actuator by 1.5 mm, which is 4.5% of the contraction of the active string actuator without the optical fiber. This reduction owes to the stiffness of the optical fiber and friction between the fiber and the active string; however, the degree of reduction was small.
2. The sensor voltage acquired using the embedded optical fiber sensor changed with the length of the active string actuator. This suggests that it is possible to estimate the displacement of the active string actuator using the optical fiber sensor.

A thinner optical fiber with a low stiffness will be integrated in a future study to improve the reduction in the contraction characteristics, and the optimal composite pitch of the optical fiber will be determined. In addition, displacement control experiments will be conducted to demonstrate the usefulness of optical fiber sensor.

Although the effect of temperature was not investigated in this study, all of the experiments were carried out at room temperature (20 °C), and the sensor values were found to be stable. In the future, we would like to study the effect of temperature. In addition, the optical fiber contacted with thin artificial muscles, so the possibility of microbending losses cannot be ruled out. However, as the optical fiber was not fixed to thin artificial muscles, and there were gap spaces between the thin artificial muscles, when the active string contracted, the fiber escaped into the gap; therefore, high stress was not added to the fiber. In addition, thin artificial muscles had a very soft structure and the stress on the optical fiber was small. Therefore, the microbending losses are considered sufficiently small. In this study, the focus was on bending losses, but in future work, we would like to examine the influence of microbending losses using fiber Bragg grating (FBG).

## Data Availability

Not applicable.
